# Methylated HBHA Produced in *M. smegmatis* Discriminates between Active and Non-Active Tuberculosis Disease among RD1-Responders

**DOI:** 10.1371/journal.pone.0018315

**Published:** 2011-03-29

**Authors:** Giovanni Delogu, Teresa Chiacchio, Valentina Vanini, Ornella Butera, Gilda Cuzzi, Alessandra Bua, Paola Molicotti, Stefania Zanetti, Francesco Nicola Lauria, Susanna Grisetti, Nicola Magnavita, Giovanni Fadda, Enrico Girardi, Delia Goletti

**Affiliations:** 1 Istituto di Microbiologia, Università Cattolica del Sacro Cuore, Roma, Italy; 2 Translational Research Unit, Department of Epidemiology and Preclinical Research, L. Spallanzani National Institute for Infectious Diseases (INMI), Rome, Italy; 3 Department of Biomedical Sciences, University of Sassari, Sassari, Italy; 4 Division of Infectious Diseases of the Respiratory Tract, Department of Clinical Research, INMI, Rome, Italy; 5 Third Division of the Clinic, Department of Clinical Research, INMI, Rome, Italy; 6 Istituto di Medicina del Lavoro, Università Cattolica del Sacro Cuore, Roma, Italy; 7 Department of Epidemiology and Preclinical Research, INMI, Rome, Italy; University of Delhi, India

## Abstract

**Background:**

A challenge in tuberculosis (TB) research is to develop a new immunological test that can help distinguish, among subjects responsive to QuantiFERON TB Gold In tube (QFT-IT), those who are able to control Mtb replication (remote LTBI, recent infection and past TB) from those who cannot (active TB disease). IFN-γ response to the Heparin-binding-hemagglutinin (HBHA) of Mtb has been associated with LTBI, but the cumbersome procedures of purifying the methylated and immunological active form of the protein from Mtb or *M. bovis Bacillus Calmette et Guerin* (BCG) have prevented its implementation in a diagnostic test. Therefore, the aim of the present study was to evaluate the IFN-γ response to methylated HBHA of Mtb produced in *M. smegmatis* (rHBHAms) in individuals at different stages of TB who scored positive to QFT-IT.

**Methodology/Principal Findings:**

87 individuals at different stages of TB who scored positive to QFT-IT were selected. IFN-γ response to *in vitro* whole blood stimulation with rHBHAms was evaluated by short-term and long-term tests and detected by ELISA or flow cytometry. We demonstrated that the IFN-γ response to rHBHAms is mediated by CD4^+^ T-cells with an effector-memory phenotype. This response, evaluated by short-term-tests, is significantly lower in active TB than in remote LTBI (p = 0.0010) and past TB (p = 0.0152). These results were confirmed by long-term tests. The qualitative data confirmed that IFN-γ responses higher than the cut-off point identified by ROC analysis are associated with the status of non-active disease.

**Conclusions:**

In this study we show that the T-cell response to a recombinant and methylated HBHA of Mtb produced in *M. smegmatis* is useful to discriminate between active and non-active TB disease among those responsive to QFT-IT in a whole blood system. Further studies are needed to improve the accuracy of the assay.

## Introduction

Tuberculosis (TB) remains a major global health problem and is one of the leading causes of morbidity and mortality due to infection (www.who.int/tb/publications/global_report/en/) [1].

The identification of biomarkers of protection and disease may be helpful for a better understanding of TB pathogenesis and eventually for diagnostic purposes. A useful approach to identify such markers could be to compare the immune responses likely to be associated with protection in infected non-diseased subjects, such as subjects with latent TB infection (LTBI), with those associated in diseased patients (subjects with active TB) [2], [3].

Recently, the introduction of T-cell-based interferon (IFN)-γ release assays (IGRAs), using antigens belonging to *Mycobacterium tuberculosis* (Mtb) region of difference (RD)1 [including early secreted antigenic target (ESAT)-6 and culture filtrate protein 10 (CFP)-10], have made a significant step towards improved LTBI diagnosis [4]. However, these tests do not discriminate between active disease, remote LTBI, recent infection and past cured TB [5]–[8].

It has previously been shown that the heparin-binding hemagglutinin (HBHA) of the MTB complex is a major latency antigen associated to LTBI [9]–[11] as assessed in PBMC by measuring IFN-γ after 4 days of stimulation. It was demonstrated that the low HBHA-induced IFN-γ production in patients with active TB [12], [13] depended upon the suppressive capacity of the T- regulatory cells in the periphery [9]. Nevertheless, no T- regulatory specific suppression was found at the site of TB disease and consequently a high local response to HBHA was detected [14]. The different HBHA-specific immune response in LTBI subjects vs. active TB patients was also observed in studies where the humoral response against HBHA was measured [10], [15]–[17], confirming that HBHA is an important antigen during Mtb infection and may be a useful biomarker to discriminate between LTBI and active TB.

Recombinant HBHA produced in *Escherichia coli* is not immunogenic and methylation of HBHA is required for the full immunological properties of the protein [10], [11], [15], [18]. To overcome the cumbersone procedures involved in the purification of native HBHA (nHBHA) [19], [20] a recombinant *M. smegmatis* strain expressing the histidine-tagged recombinant HBHA protein from Mtb (rHBHAms) was developed and used to purify a large amount of protein [21]. The methylation pattern of rHBHAms was similar to that observed for nHBHA (rHBHAms ≈16 methyl groups vs ≈23 methyl groups in nHBHA [22], as assessed by mass spectrometry analysis, Delogu G. unpublished), and this partial methylation was shown to be sufficient to rescue the immunological properties of HBHA as shown in humoral response studies [15]–[17].

Therefore, the aim of the present study was to evaluate tools that may help to discriminate the different phases of TB among those positive to an IGRA, i.e the QuantiFERON TB Gold In tube (QFT-IT), due to remote LTBI, recent infection, past TB, or active TB. In these subjects we characterized the T-cell-specific immune response to a recombinant and methylated HBHA produced in *M. smegmatis* (rHBHAms) by short and long-term whole blood stimulation and cytometry.

## Results

### Characteristics of the population

We studied 24 individuals with remote LTBI, 19 with recent TB infection, 18 with past TB and 26 with active TB. Median age, gender, origin, BCG vaccination status and QFT-IT are reported among the different groups (Table 1). A significant difference among the different groups was found in terms of origin (p<0.0001) and BCG vaccination status (p<0.0001). By “origin” we mean the place where the enrolled individuals were born. At the time of enrollment they all lived in Italy where they had been living for at least one year (median: 6 years, IQR 1–22 years).

**Table 1:** Demographic and clinical characteristics of the subjects enrolled in the study.

	TotalN (%)87 (100.0)	Remote infectionN (%)24 (27.6)	Recent infectionN (%)19 (21.8)	Past TBN (%)18(20.7)	Active TBN (%)26 (29.9)	Pvalue
**Median Age** **(IQR)**	39(27–50)	48(30.5–59)	39(28–50)	39(28.5–50.7)	35(25.–44.2)	0.295
**Male Gender**	53 (60.9)	12 (50.0)	10 (52.6)	13(72.2)	18 (69.2)	0.325
**Origin**						<0.0001
**Eastern Europe**	33 (37.9)	3 (12.5)	4 (21.1)	11 (61.1)	15(57.7)	
**Western Europe**	39 (44.8)	21 (87.5)	8 (42.1)	5 (27.8)	5 (19.2)	
**Africa**	2 (2.3)	-	1 (5.3)	-	1 (3.8)	
**Asia**	5 (5.7)	-	1 (5.3)	1 (5.6)	3 (11.5)	
**South America**	8 (9.2)	-	5 (26.3)	1 (5.6)	2 (7.7)	
**BCG**						<0.0001
**Vaccinated**	32 (36.8)	2 (8.3)	9 (47.4)	8 (44.4)	13 (50.0)	
**Unvaccinated**	41 (47.1)	20 (83.3)	7 (36.8)	9 (50.0)	5 (19.2)	
**NA**	14 (16.1)	2 (8.3)	3 (15.8)	1 (5.6)	8 (30.8)	
**QTF-IT**						NA
**Positive**	87 (100.0)	24 (100.0)	19 (100.0)	18 (100.0)	26 (100.0)	
**Negative**	-	-	-	-	-	

**Footnotes: TB: tuberculosis; IQR: interquartile range; BCG: Bacillus Calmètte et Guerin; QFT-IT:QuantiFERON TB Gold In Tube; NA: not available.**

### IFN-γ dose-dependent response to rHBHAms

**Figure 1:**
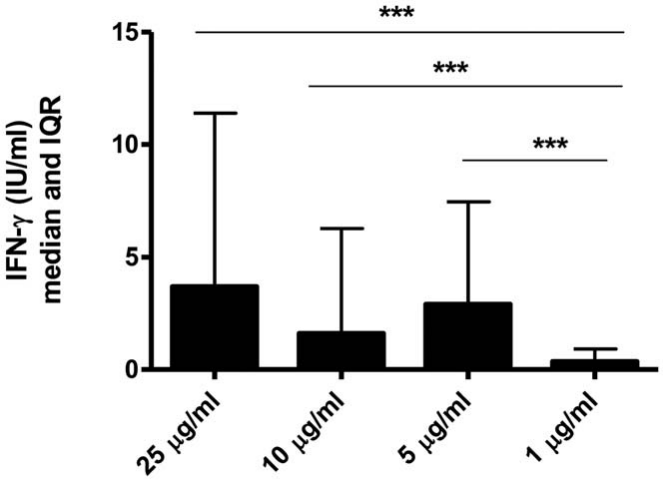
Concentration-dependent IFN-γ response after *in vitro* short-term whole blood stimulation with rHBHAms in subjects with LTBI. Whole blood from 10 subjects with LTBI was stimulated with or without rHBHAms at different concentrations (between 25 and 1 µg/ml). IFN-γ response was evaluated after a short-term stimulation (1 day post-*in vitro* stimulation). A significant difference was found for the IFN-γ response obtained between 25 and 5 µg/ml and that obtained at a concentration of 1 µg/ml.

To determine the most appropriate concentration of rHBHAms to be used *in vitro*, 10 subjects with LTBI were evaluated, and the IFN-γ-specific response following *in vitro* short-term whole blood stimulation with rHBHAms was assessed. As shown in Figure 1, a significant difference was found between the IFN-γ responses when comparing rHBHAms at a concentration of 1 µg/ml to concentrations of 25 µg/ml (p = 0.002), 10 µg/ml (p = 0.008) or 5 µg/ml (p = 0.004). No significant differences were observed for the other pair-wise comparisons. Therefore, from hereinafter the concentration of rHBHAms used in these *in vitro* assays was 5 µg/ml.

### Short-term IFN-γ response to different antigens: quantitative analysis

#### Response to QFT-IT

**Figure 2:**
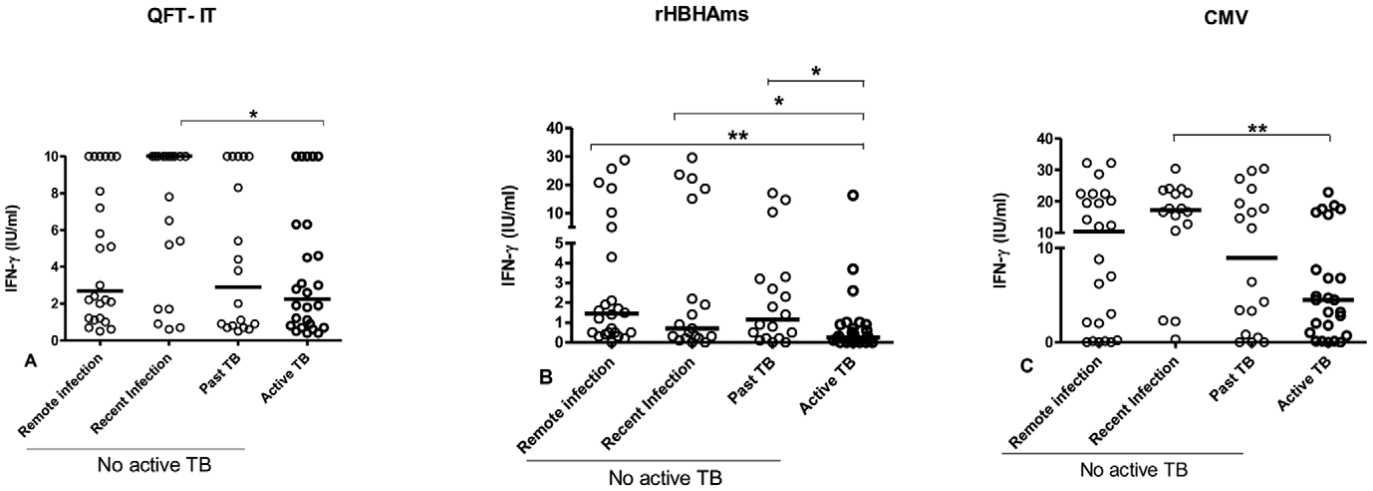
Response to rHBHAms is significantly impaired in patients with active TB. Whole blood was stimulated with or without QFT-IT (**A**), rHBHAms (**B**) and CMV lysate (**C**). IFN-γ response was evaluated after a short-term stimulation (1 day post-*in vitro* stimulation). The data are shown after the subtraction of the results obtained in the unstimulated sample. A significant difference in terms of IFN-γ release to QFT-IT (**A**) and CMV lysate (**C**) was found between those with active TB and recent infection (p = 0.01 and p = 0.002 respectively). A significant lower response to rHBHAms (**B**) was found in patients with active TB compared to those with remote LTBI (p = 0.001) and past TB (p = 0.02).

All of the enrolled subjects responded to the mitogen (data not shown) and to QFT-IT by the selection criteria (see materials and methods) and categorized as remote LTBI, recent infection, past TB and active TB. No significant differences were found among the different groups in response to QFT-IT, except between those with a recent infection (median: 10.0 IU/ml; IQR: 1.7–10.0) and those with active TB (median: 2.25 IU/ml; IQR: 0.77–6.30) (p = 0.01) (**Figure 2A**).

#### Response to rHBHAms

IFN-γ production in response to the rHBHAms was significantly lower in those with active TB (median: 0.2 IU/ml, IQR: 0–16.3) compared to those with remote LTBI (median: 1.4 IU/ml, IQR: 0–28.7) (p = 0.001) and subjects with past TB (median: 1.1 IU/ml, IQR: 0–17.0) (p = 0.02) (**Figure 2B**), and not significantly different compared to those with a recent infection (0.7 IU/ml (IQR: 0–29.5) (p = 0.052). No significant differences were found among the different groups without active TB (remote LTBI, recent infection, past TB).

#### Response to Cytomegalovirus (CMV) lysate

As an internal control, we evaluated the IFN-γ production in response to the recall antigen CMV. Response to CMV was analyzed in all subjects with remote LTBI and past TB and a large portion of the subjects from the other groups (16/19 of the recent infection group; 25/26 of the active TB group). IFN-γ response to CMV was significantly different only between those with a recent infection (median: 17.2 IU/ml; IQR 0.3–30.4) and those with active TB (median: 4.5 IU/ml; IQR 0–22.9) (p = 0.002). (**Figure 2C**).

All together, these data suggest that among those who scored positive to QFT-IT, the IFN-γ response to rHBHAms was more frequently found in those able to control *Mtb* infection, either naturally (remote LTBI and recent infection) or after chemotherapy (past TB) than in those with an ongoing *Mtb* replication (active disease). Noteworthy, a down regulation of the response to rHBHAms was observed in active TB patients compared to those with past cured TB.

### *In vitro* short-term and long-term IFN-γ responses to rHBHAms and QFT-IT: ROC analysis and qualitative evaluations

**Figure 3:**
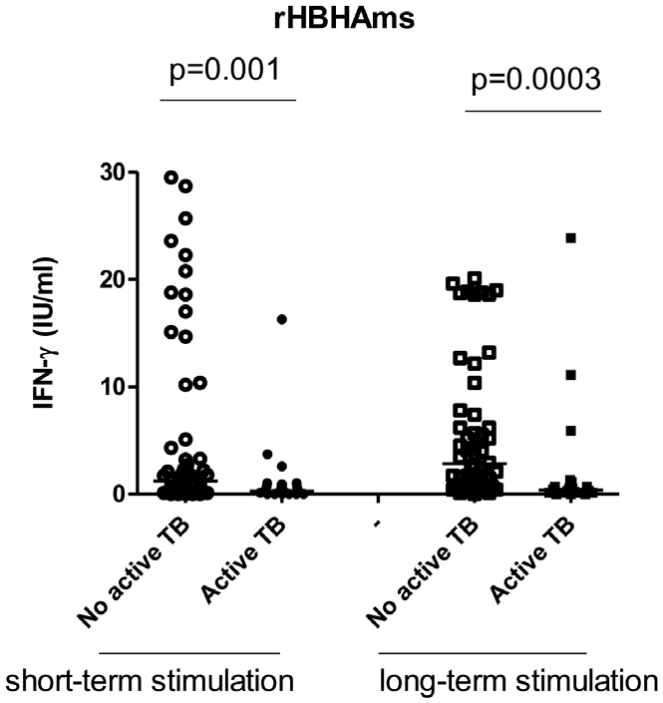
Response to rHBHAms is significantly impaired in patients with active TB after short- or long- term stimulation. A significant difference was found for the IFN-γ response to rHBHA between the subjects with and without active TB (p = 0.001) evaluated by short- or long- term stimulation.

**Figure 4:**
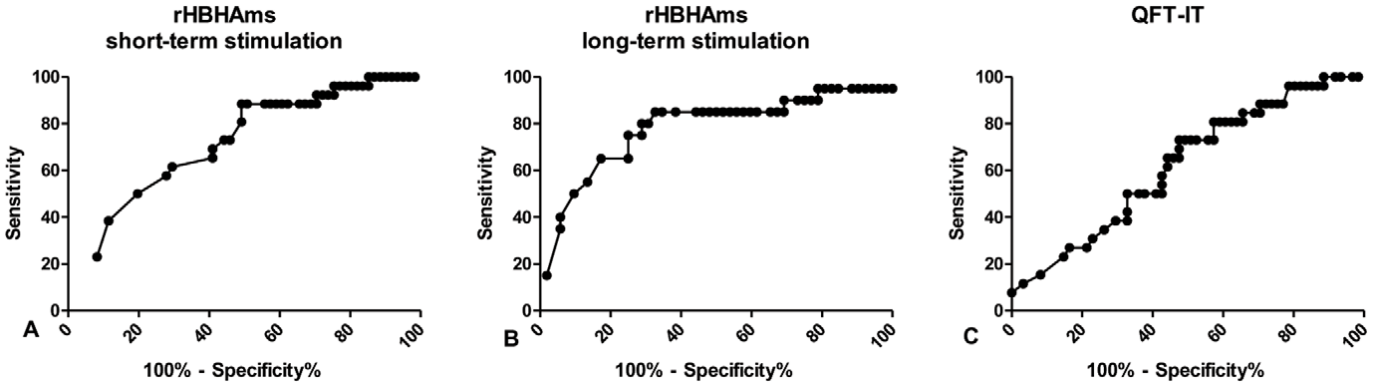
IFN-γ response to rHBHAms in short- and long-term-“*in vitro*” stimulation and to QFT-IT: ROC analysis. A ROC analysis for the response to rHBHAms obtained in whole blood by short-term stimulation (**A**), long- term stimulation (**B**) and QFT-IT (**C**) was performed using the active TB patients and the subjects without active TB as comparator groups.

Because no differences were found among the subjects without active TB for the short-term response to rHBHAms (remote LTBI, recent infection, past TB), the data from these groups were pooled together and indicated as results from “no active TB” subjects. A significant difference was found for the response to rHBHA between the subjects with and without active TB (p = 0.001) (**Figure 3**). Therefore, based on this result, we performed a ROC analysis to evaluate the potential use of this assay for discriminating the different stages of TB. We found significant results in the AUC analysis (AUC, 0.72; 95% confidence interval (CI), 0.60–0.83, p = 0.001) (**Figure 4A**). For scoring purposes, a cut-off value was chosen to maximize the sum of sensitivity and specificity. The use of a cut-off point below 0.25 IU/ml showed 50.0% sensitivity (95% CI, 29.93%–77.89%) and 80.3% specificity (95% CI, 68.16%–89.40) to identify those with active TB. Therefore, based on this cut-off value, we scored the results as negative and positive and a pair-wise comparison was performed. A significantly lower proportion of positive results was found among those with active TB (13/26; 50%) compared to those without (12/61; 80.3%) (p = 0.0008).

To assess the memory response, we evaluated the long-term IFN-γ production to rHBHAms in the majority of the subjects enrolled (20/26 with active TB and 52/61 without) and a significant difference was found between these two groups (p = 0.0003) (**Figure 3**). Therefore, based on this data, a ROC analysis was performed and we found significant results for the AUC analysis (AUC, 0.78; 95% confidence interval (CI), 0.64–0.91, p = 0.0002) (**Figure 4B**). For scoring purposes we chose a cut-off point to maximize the sum of sensitivity and specificity. The use of a cut-off point below 0.75 IU/ml showed 75.0% sensitivity (95% CI, 50.90%–91.34%) and 75.0% specificity (95% CI, 61.05%–85.97) to identify those with active TB. Therefore, based on this cut-off point, we scored the results as negative and positive and a pair-wise comparison was performed. A significantly lower proportion of positive results was found among those with active TB (5/20; 25%) compared to those without (39/52; 75.0%) (p = 0.0003).

In the same subjects, we also investigated whether the response to QFT-IT could be useful to discriminate the different stages of TB. No significant results for the area under the curve (AUC) analysis were obtained (AUC, 0.62; 95% confidence interval (CI), 0.49–0.74, p = 0.07) (**Figure 4C**) and therefore no further statistical analysis was performed.

All together, these data suggest that among those who scored positive to QFT-IT, the IFN-γ response to rHBHAms was more frequently found in those able to control Mtb infection, than in those with an ongoing Mtb replication as evaluated by both short and long-term whole blood assays.

### Memory response to rHBHAms is impaired in those with active TB

**Figure 5:**
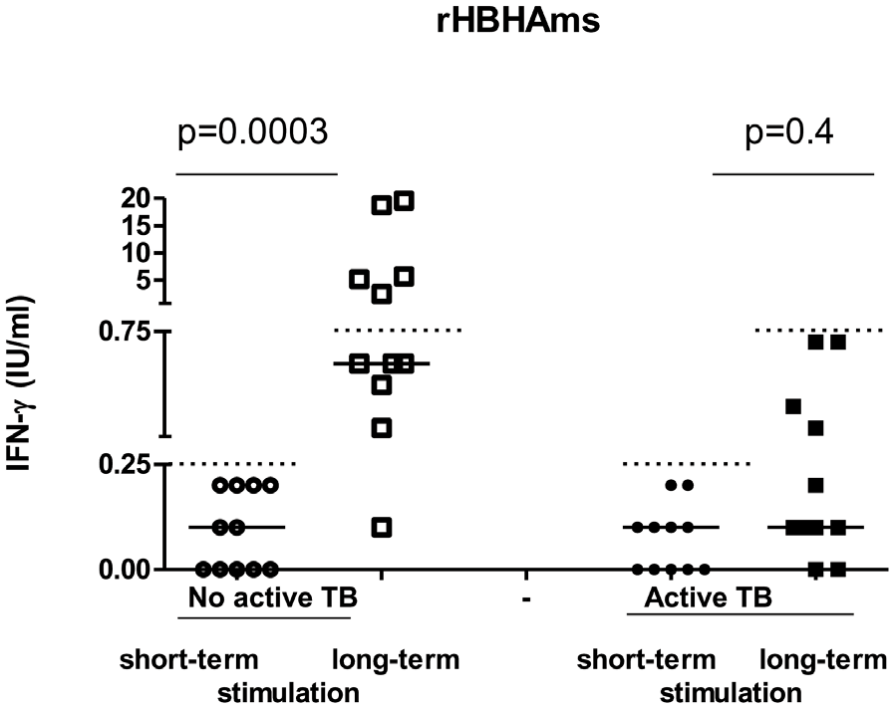
Memory responses to rHBHAms. Memory response (long-term stimulation) to rHBHAms was evaluated in the subjects who scored negative on the short- test. A significant difference was found between the IFN-γ value obtained in the short-term stimulation compared to the long-term stimulation among those without active TB (p = 0.0003). Differently, no significant difference was found between the short- and long- term stimulation among those with active TB (p = 0.4). The data are shown after the subtraction of the results obtained in the unstimulated samples. Dotted lines indicate the cut-off obtained for the short- and long-term tests.

To further assess the memory response we evaluated the long-term IFN-γ production to rHBHAms in a subgroup of the subjects who scored negative (below 0.25 IU/ml) to this antigen on the short-term test (11 without active disease and 11 with active TB). As shown in **Figure 5**, a significant difference was found between the IFN- γ value by the short-term test (median: 0.1 IU/ml; IQR 0–0.2) and the long-term test (median: 0.6 IU/ml; IQR 0.5–5.7) (p = 0.0003) among those without active TB. Conversely, among those with active TB, no significant IFN-γ difference was found when the short-term test (median: 0.1 IU/ml; IQR 0–0.1) was compared to the long-term test (median: 0.1 IU/ml; IQR 0.1–0.4) (p = 0.4). Based on the previously established cut-off values (**Figure 4**), a significantly higher proportion of memory response was found in the subjects without active disease (5/11, 45.4%) compared with those with active TB (0/11, 0%) (p = 0.03) (**Figure 5**). All together, these data indicate that among those with active TB, the recovery of a memory response to rHBHAms is unlikely.

### IFN-γ in response to rHBHAms is mediated by CD4^+^ effector memory T lymphocytes

**Figure 6:**
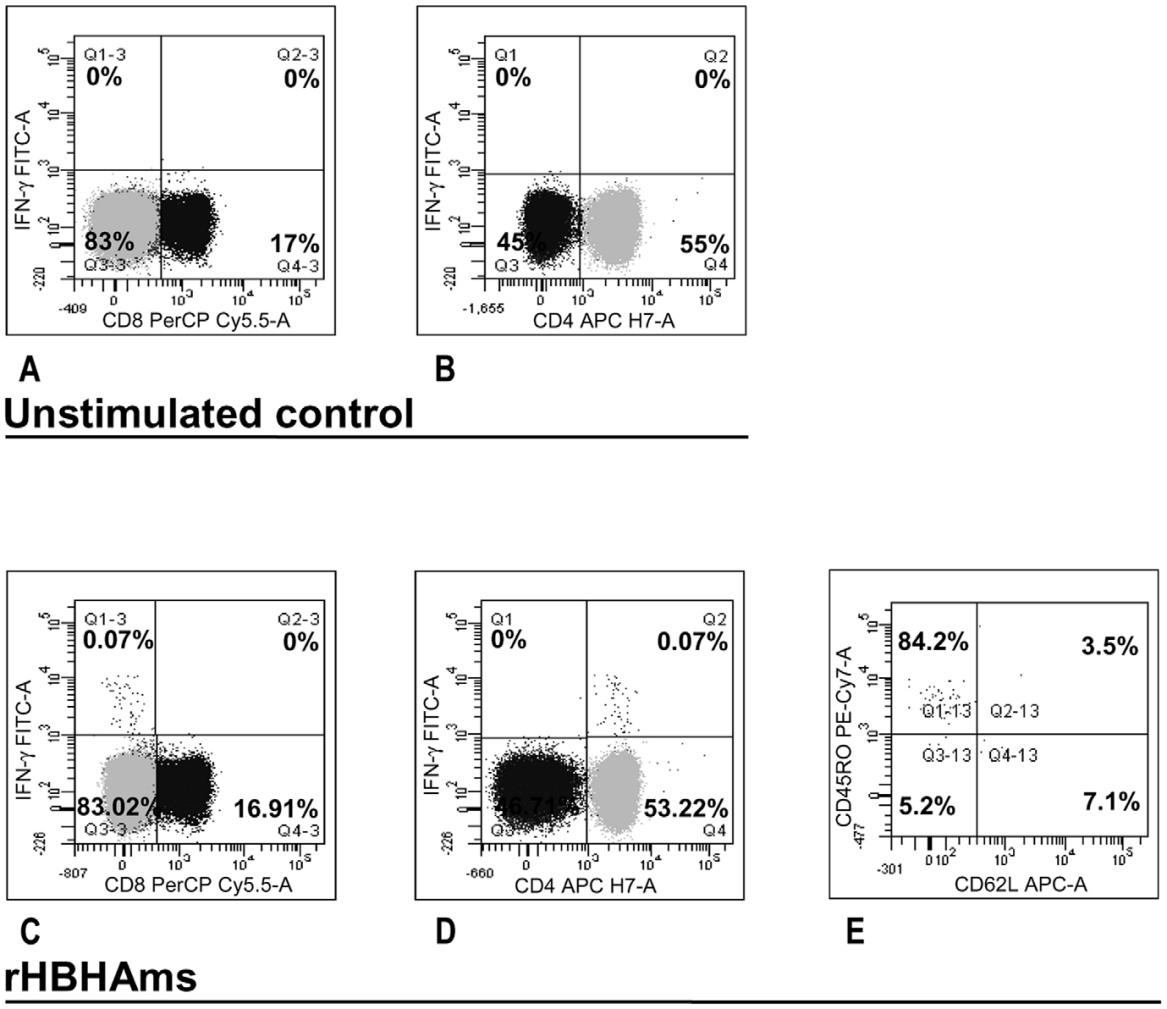
CD4^+^ effector memory T lymphocytes produce IFN-γ in response to rHBHAms. The phenotypic characteristics of the cells responding to the rHBHAms in the HBHA-responders were evaluated. As shown in a representative subject, a significant IFN-γ response to the rHBHAms was observed for CD4^+^ T-cells (**D**) over the negative control (**B**), whereas no response was detected for CD8^+^ T-cells (**C**) over the negative control (**A**). To characterize this immune response, naive and memory phenotypes were studied. Most of the CD4^+^ T-cells IFN-γ responding to the rHBHAms presented an effector memory phenotype (84%) defined as CD45R0^+^CD62L (**E**).

We further evaluated the phenotypic characteristics of the cells responding to the rHBHAms in the HBHA-responders in the short-term test. All the subjects analyzed responded to the positive control, PMA plus IONO (data not shown). As shown in a representative subject in **Figure 6**, a significant IFN-γ response to the rHBHAms was observed for CD4^+^ T-cells (**Figure 6D**) over the negative control (**Figure 6B**), whereas no response was detected for CD8^+^ T-cells (**Figure 6C**) over the negative control (**Figure 6A**). To characterize this immune response, naive and memory phenotypes were studied. Most of the CD4^+^ T-cells IFN-γ responding to the rHBHAms presented an effector memory phenotype (84%) defined as CD45R0^+^CD62L (**Figure 6 E**).

All together, these data suggest that the response to rHBHAm is mediated by CD4^+^ T-cells with an effector-memory phenotype.

## Discussion

A major challenge in TB research is to develop a new immunological test that can help distinguish, among subjects responsive to QFT-IT, those who are able to control Mtb replication (subjects with remote LTBI, recent infection and past TB) from those who cannot (patients with active TB disease). This discrimination would be very useful because it would allow for targeting those needing a rapid full course therapy and thus avoid dissemination of the infection in the community.

In this study, we show that although patients with active TB respond to QFT-IT and to another non-Mtb recalling antigen such as CMV, the IFN-γ response to the rHBHAms was almost absent. Moreover, no recovery of a memory response to rHBHAms was found in these patients, different from the results found using other Mtb-specific recalling antigens [23], [24]. For the first time to our knowledge we showed that the recombinant and methylated HBHA of Mtb produced in *M. smegmatis*, which can be easily and inexpensively produced, is immunogenic and potentially useful to exclude active TB in a T-cell based in vitro system. Therefore, by combining the QFT-IT score with the rHBHAms results, we identified a potential two-step immune approach for the identification of subjects at different stages of TB. Subjects responding to both antigens are more likely to be able to control Mtb replication (such as those with remote LTBI, recent infection or past TB), while those responding only to QFT-IT are more likely to present active TB.

In previous studies using a 4-day culture of PBMC [10], it was shown that the IFN-γ response to native HBHA, purified from *M. bovis* BCG, is associated with LTBI, whereas this response is almost lost during active TB, due to the specific suppression mediated by the regulatory T-cells [9]. Therefore the assay based on IFN-γ in response to HBHA has been proposed either to discriminate LTBI subjects from patients with active TB or to increase the sensitivity and specificity of RD1-based IGRAs [13]. However, since orthologs of HBHA have been identified in other mycobacterial species, including environmental mycobacteria [20] and BCG, and consequently positive IFN-γ responses to HBHA have been found in BCG-vaccinated subjects [12], the specificity of an HBHA-based assay was questioned.

In the present study, we implemented a potential diagnostic algorithm that relies on the well-documented ability of the QFT-IT to identify Mtb infection [25]. Therefore among those positive to QFT-IT, we used HBHA as a tool to distinguish the different stages of TB. The results indicate that the accuracy of the long-term test, that mostly measures a memory response [23], was greater compared to that reported for the short-term test that measures mostly the effector response, as shown here by flow cytometry data. Based on these results, the long-term whole blood assay may be more accurate for identifying those with active disease (positive response to QFT-IT, negative response to rHBHAms). Moreover, we showed that BCG vaccination did not have any impact on the IFN-γ response to HBHA. In fact individuals with active TB and past TB, although presenting a similar proportion of BCG-vaccinated individuals (table 1), showed a significantly different IFN-γ response to HBHA (Figure 2B).

The use of HBHA as a biomarker in TB could be crucial, for instance in patients with active TB disease, to predict durable (non-relapsing) treatment success or in subjects with LTBI to assess reactivation risk [26]. This could be of paramount importance in populations that are at a higher risk of developing active TB, such as HIV-positive subjects, in communities with a high TB prevalence [27] , subjects with rheumatoid arthritis [5], or patients for whom diagnosis of active TB and monitoring or treatment may be difficult, such as pediatric patients [26]. Further studies specifically addressing these questions will provide a clearer picture on the usefulness of the QFT-IT/rHBHAms combination assay to identify different TB status.

A major finding of this study was also that the T-cell response against HBHA is quantitatively and qualitatively different depending on the stages of infection. This is remarkable since we could measure a quantitative difference in the QFT-IT response between the group of the recently infected subjects and the active TB patients, but not in the other groups investigated. Similar results were obtained using CMV lysate, a non-Mtb specific antigen, suggesting that the difference obtained by analyzing the QFT-IT data was not Mtb-specific. Conversely, the secretion of IFN-γ induced by rHBHAms was significantly lower in the group of patients with active TB and was rescued following successful anti-TB therapy, as shown by the results obtained in the group of subjects with past cured TB. This switch in the T-cell response specifically directed against HBHA epitopes was observed by assays that measure both effector and memory response (1- day and 7- day tests) [23], indicating that the emergence of TB disease correlates with a significant change in the HBHA-specific immune response. It would be of interest to understand whether the modulation of HBHA expression by the bacilli during infection can induce this change in the host response and promote disease progression, as studies in animal models suggest [28] or whether the switch that we observed was an effect independent from the HBHA role in pathogenesis.

The classical dichotomy between active TB and LTBI is being reconsidered in favor of a continuous and dynamic spectrum of conditions extending from infection to disease [2], [27]. This spectrum of TB results from the interaction between the host immune response and the pathogen and can be maintained in a dynamic and variable equilibrium for decades. Moreover, in the same TB patients, diverse lesions ranging from sterility to multi-bacillary disease have been observed, suggesting that the entire spectrum of TB can coexist in the same individual [2], [27], [29]. In this scenario, the observation that the host immune response against a single antigen (HBHA) is dependent on the specific TB status is of the utmost importance [3]. The results of this study highlight the potential usefulness of rHBHAms as a biomarker for TB in association with the RD1-based IGRAs. rHBHAms can be obtained through a simplified protocol, therefore, if these data are confirmed in larger , blinded studies, their implementation in the current clinical laboratory settings can become feasible and economic. In conclusion, for the first time to our knowledge, we showed that the T-cell based response to a recombinant and methylated HBHA of Mtb produced in *M. smegmatis* in a whole blood system is immunogenic and potentially useful to discriminate active from non-active disease. Further studies are needed to improve the accuracy of the assay and to prove its reproducibility.

## Materials and Methods

### Study population

The study was approved by the Ethical Committee of the National Institute of Infectious diseases L. Spallanzani (“Parere 18/2002”, INMI) and all enrolled individuals provided written informed consent. Upon enrollment, demographic and epidemiological information was collected through a structured questionnaire.

The following individuals were enrolled: 1) “remote LTBI”, individuals who scored positive to TST and QFT-IT and reported household or equivalent close contact with smear-positive pulmonary TB patients in the 3 years before enrolment; 2) “recent infection”, individuals who reported household or equivalent close contact with smear-positive pulmonary TB patients in the previous 3 months, scored positive to TST and QFT-IT but who had not yet started a specific prophylaxis; 3)“active TB”, individuals diagnosed either by a positive culture for Mtb from sputa or with a positive Mtb-specific RNA amplification (MTD Test, Gen-probe, San Diego, USA) from biopsy specimens and/or biological fluids) who started specific treatment within a month and scored positive to QFT-IT; 4) “past TB”, individuals with documented culture-positive pulmonary TB who successfully completed anti-TB treatment (culture-negative upon treatment completion), who were evaluated from 6 months up to 2 years after treatment and had scored positive to QFT-IT at the time of the study.

Individuals who tested positive to a human immunodeficiency virus (HIV) antibody test or were on immunosuppressive drugs were not included in the study. Subjects with underlying immune-mediated disease were also excluded.

### Purification of the methylated HBHA

Recombinant methylated HBHA was purified from *M. smegmatis* pMV3-38 as previously described [21]. Briefly, the *M. smegmatis* pMV3-38, expressing the histidine-tagged, full length HBHA under the control of the *hbh*A promoter, was grown in Sauton media for three days until late log phase was reached. Cells were harvested by centrifugation, lysed by sonication and the cell lysate was subjected to Nickel chromatography (X-Press, Invitrogen). The fractions containing the purified protein were dialysed in phosphate-buffered saline pH 7.0 (PBS) and concentrated using Amicon Centricon Centrifugal filter devices (Millipore, Beverly, MA USA). Batches of purified protein of 1 mg/ml were stored at −80°C until used.

### TST

TST was administered by the Mantoux procedure using 5 IU of purified protein derivative (Chiron, Siena, Italy). Results were read after 72 hrs. Indurations of at least 5 mm or ≥10 mm were scored as positive for close contacts or for the other conditions, respectively [30] .

### Whole blood enzyme linked immunosorbent assays with rHBHAms

#### 1-day (short-term) response

0.5 ml per well of heparinised whole blood was seeded in a 48-well plate (Corning Costar, Corning Incorporated, New York, NY, USA) and treated with phytohaemagglutinin (PHA) at 5 µg/ml (Sigma, St Louis, MO, USA), rHBHAms at 5 µg/ml unless differently indicated, and human CMV lysate antigen from the strain AD 169, at 5 µg/ml (Advanced Biotechnologies Inc. Columbia, MD). Samples were then incubated for 20–24 hrs; supernatant was then harvested and stored at −20°C until tested.

#### 7-day (long-term) response

We used the previously described methodology [23]. Briefly, on the day of blood collection, an aliquot of heparinised blood was diluted 5-fold using RPMI 1640 supplemented with penicillin, streptomycin and 2 mM L-glutamine (the last four products are from Euroclone Ltd, United Kingdom), plated into 48-well plates (Corning Costar) and stimulated as described above. The day-7 diluted supernatant was harvested following incubation at 37°C and stored at −20°C until tested.

The persons performing the assays were blinded to the clinical status of the samples being tested.

### IFN-γ determination

IFN-γ from day-1 and day-7 supernatant was evaluated by a commercial ELISA (CMI, Cellestis Limited, Carnegie, Victoria, Australia) and data were presented as IU/ml after subtraction of the appropriate control.

### Commercially available IGRA

QuantiFERON TB-Gold In tube (QFT-IT) (Cellestis Limited) was performed and its results were scored as indicated by the manufacturer (the cut-off value for a positive test was 0.35 IU/ml).

### Phenotypic and functional FACS analysis

The phenotypic and functional analysis of peripheral whole blood cells was performed by flow cytometry after overnight culture in complete medium in the presence or absence of the following stimuli: phorbol-12-myristate-13-acetate (PMA) at 3 nM (SERVA Electrophoresis GmbH, Heidelberg, Germany) plus ionomycin (IONO) at 1,5 µM (SERVA) used as positive controls and rHBHAms at 5 µg/ml. Expression of different markers was assessed by staining with appropriate combinations of monoclonal antibodies (MoAb) directly conjugated to fluorochromes: allophycocyanin (APC)-H7-conjugated anti-CD4; peridinin chlorophyll-protein (PerCP)-Cy5.5-conjugated anti-CD8; phycoerythrin (PE)-Cy7-conjugated anti-CD45RO, allophycocyanin (APC)-conjugated anti-CD62L (all from Becton Dickinson (BD) Biosciences, San Jose, USA). To detect intracellular expression of cytokines, 50 µg/ml of Brefeldin A (SERVA) was used, as previously described. Briefly, production of IFN-γ was assessed by staining with appropriate combinations of MoAb conjugated directly to fluorochromes [fluorescein isothiocyanate (FITC)-conjugated anti- IFN-γ 2 (BD)]. The cells were stimulated *in vitro* for 1 day with anti-CD3 and anti-CD28 antibodies of 2 µg/ml each (BD). Data acquisition and analysis were performed on an FACS CantoII flow cytometer (BD) using FACSDiva software (version 6.1.2; BD). For all staining procedures, an isotype-matched negative control was processed in parallel.

### Statistical analysis

The main outcome of the study was the evaluation of IFN-γ production in response to antigenic stimulation, expressed as continuous (IU/ml) or dichotomous (positive/negative) measures. The median and IQR of IFN-γ production were calculated; the Mann-Whitney U test was used to compare medians for pair-wise comparisons; the Kruskal-Wallis test was used to compare medians among the different groups. The cut-off value for scoring purposes was defined by a receiver-operator characteristic analysis (ROC); the chi square was used for dichotomous measures. SPSS v 14 for Windows (SPSS Italia Srl, Bologna, Italy) and Prism 4 software (Graphpad Software 4.0, San Diego, CA, USA) were used in the analysis. Differences were considered significant at p values ≤0.05. In this study we used the term sensitivity for the detection of the “condition having active TB” when we measured the proportion of actual individuals with active disease who were correctly identified as such. We used the term “specificity” for the detection of the “condition not having active TB” when we measured the proportion of those without active TB who were correctly identified as such.

## References

[1] World Health Organization (2010). Global Tuberculosis Control. Surveillance, Planning, Financing.. http://www.who.int/tb/publications/global_report/en/.

[2] Barry CE, Boshoff HI, Dartois V, Dick T, Ehrt S (2009). The spectrum of latent tuberculosis: rethinking the biology and intervention strategies.. Nat Rev Microbiol.

[3] Pai M (2010). Spectrum of latent tuberculosis - existing tests cannot resolve the underlying phenotypes.. Nat Rev Microbiol.

[4] Mack U, Migliori GB, Sester M, Rieder HL, Ehlers S (2009). LTBI: latent tuberculosis infection or lasting immune responses to M. tuberculosis? A TBNET consensus statement.. Eur Respir J.

[5] Solovic I, Sester M, Gomez-Reino JJ, Rieder HL, Ehlers S (2010). The risk of tuberculosis related to tumour necrosis factor antagonist therapies: a TBNET consensus statement.. Eur Respir J.

[6] Goletti D, Butera O, Vanini V, Lauria FN, Lange C (2010). Response to Rv2628 latency antigen associates with cured tuberculosis and remote infection.. Eur Respir J.

[7] Goletti D, Carrara S, Butera O, Amicosante M, Ernst M (2008). Accuracy of immunodiagnostic tests for active tuberculosis using single and combined results: a multicenter TBNET-Study.. PLoS ONE.

[8] Goletti D, Parracino MP, Butera O, Bizzoni F, Casetti R (2007). Isoniazid prophylaxis differently modulates T-cell responses to RD1-epitopes in contacts recently exposed to Mycobacterium tuberculosis: a pilot study.. Respir Res.

[9] Hougardy JM, Place S, Hildebrand M, Drowart A, Debrie AS (2007). Regulatory T cells depress immune responses to protective antigens in active tuberculosis.. Am J Respir Crit Care Med.

[10] Masungi C, Temmerman S, Van Vooren JP, Drowart A, Pethe K (2002). Differential T and B cell responses against Mycobacterium tuberculosis heparin-binding hemagglutinin adhesin in infected healthy individuals and patients with tuberculosis.. J Infect Dis.

[11] Temmerman S, Pethe K, Parra M, Alonso S, Rouanet C (2004). Methylation-dependent T cell immunity to Mycobacterium tuberculosis heparin-binding hemagglutinin.. Nat Med.

[12] Savolainen L, Pusa L, Kim HJ, Sillanpaa H, Seppala I (2008). Pilot study of diagnostic potential of the Mycobacterium tuberculosis recombinant HBHA protein in a vaccinated population in Finland.. PLoS ONE.

[13] Hougardy JM, Schepers K, Place S, Drowart A, Lechevin V (2007). Heparin-Binding-Hemagglutinin-Induced IFN-gamma Release as a Diagnostic Tool for Latent Tuberculosis.. PLoS ONE.

[14] Place S, Verscheure V, de San N, Hougardy JM, Schepers K (2010). Heparin-binding, hemagglutinin-specific IFN-gamma synthesis at the site of infection during active tuberculosis in humans.. Am J Respir Crit Care Med.

[15] Zanetti S, Bua A, Delogu G, Pusceddu C, Mura M (2005). Patients with pulmonary tuberculosis develop a strong humoral response against methylated heparin-binding hemagglutinin.. Clin Diagn Lab Immunol.

[16] Shin AR, Lee KS, Lee JS, Kim SY, Song CH (2006). Mycobacterium tuberculosis HBHA protein reacts strongly with the serum immunoglobulin M of tuberculosis patients.. Clin Vaccine Immunol.

[17] Yang CS, Lee JS, Lee HM, Shim TS, Son JW (2008). Differential cytokine levels and immunoreactivities against Mycobacterium tuberculosis antigens between tuberculous and malignant effusions.. Respir Med.

[18] Menozzi FD, Bischoff R, Fort E, Brennan MJ, Locht C (1998). Molecular characterization of the mycobacterial heparin-binding hemagglutinin, a mycobacterial adhesin.. Proc Natl Acad Sci U S A.

[19] Menozzi FD, Rouse JH, Alavi M, Laude-Sharp M, Muller J (1996). Identification of a heparin-binding hemagglutinin present in mycobacteria.. J Exp Med.

[20] Delogu G, Brennan MJ (1999). Functional domains present in the mycobacterial hemagglutinin, HBHA.. J Bacteriol.

[21] Delogu G, Bua A, Pusceddu C, Parra M, Fadda G (2004). Expression and Purification of Recombinant Methylated HBHA in *Mycobacterium smegmatis*.. FEMS Microbiol Lett.

[22] Pethe K, Bifani P, Drobecq H, Sergheraert C, Debrie AS (2002). Mycobacterial heparin-binding hemagglutinin and laminin-binding protein share antigenic methyllysines that confer resistance to proteolysis.. Proc Natl Acad Sci U S A.

[23] Butera O, Chiacchio T, Carrara S, Casetti R, Vanini V (2009). New tools for detecting latent tuberculosis infection: evaluation of RD1-specific long-term response.. BMC Infect Dis.

[24] Lawn SD, Bangani N, Vogt M, Bekker LG, Badri M (2007). Utility of interferon-gamma ELISPOT assay responses in highly tuberculosis-exposed patients with advanced HIV infection in South Africa.. BMC Infect Dis.

[25] Pai M, Zwerling A, Menzies D (2008). Systematic review: T-cell-based assays for the diagnosis of latent tuberculosis infection: an update.. Ann Intern Med.

[26] Wallis RS, Pai M, Menzies D, Doherty TM, Walzl G (2010). Biomarkers and diagnostics for tuberculosis: progress, needs, and translation into practice.. Lancet.

[27] Lawn SD, Wood R, Wilkinson RJ (2011). Changing concepts of “latent tuberculosis infection” in patients living with HIV infection.. Clin Dev Immunol.

[28] Delogu G, Sanguinetti M, Posteraro B, Rocca S, Zanetti S (2006). The hbhA gene of Mycobacterium tuberculosis is specifically upregulated in the lungs but not in the spleens of aerogenically infected mice.. Infect Immun.

[29] Lin PL, Rodgers M, Smith L, Bigbee M, Myers A (2009). Quantitative comparison of active and latent tuberculosis in the cynomolgus macaque model.. Infect Immun.

[30] (2000). Diagnostic Standards and Classification of Tuberculosis in Adults and Children. This official statement of the American Thoracic Society and the Centers for Disease Control and Prevention was adopted by the ATS Board of Directors, July 1999. This statement was endorsed by the Council of the Infectious Disease Society of America, September 1999.. Am J Respir Crit Care Med.

